# Fisheries‐induced evolution of alternative male life history tactics in Coho salmon

**DOI:** 10.1111/eva.12970

**Published:** 2020-04-21

**Authors:** Kyle A. Young, Victoria A. Cluney, Laura K. Weir

**Affiliations:** ^1^ First Order Ecology Somerset PA USA; ^2^ Biology Department Saint Mary’s University Halifax NS Canada

**Keywords:** alternative reproductive tactics, artificial selection, fishery‐imposed selection, hooknose, jack, life history evolution, sneaker male, threshold‐dependent trait

## Abstract

Fisheries‐induced evolution (FIE) can result when harvest imposes artificial selection on variation in heritable phenotypic traits. While there is evidence for FIE, it remains difficult to disentangle the contributions of within‐generation demographic adjustment, phenotypic plasticity, and genetic adaption to observed changes in life history traits. We present evidence for FIE using dozens of Coho salmon (*Oncorhynchus kisutch*) populations in which males adopt one of two age‐invariant, heritable life history tactics: most mature as large three‐year‐old “hooknose” and typically fight for spawning opportunities, while some mature as small two‐year‐old “jacks” and fertilize eggs through sneaking. The closure of a fishery targeting three‐year‐old fish provided an experimental test of the prediction that fishery‐imposed selection against hooknose males drives an evolutionary increase in the proportion of males adopting the jack tactic. The data support the prediction: 43 of 46 populations had higher jack proportions during than after the fishery. The data further suggest that changes in jack proportion were not solely the result of demographic adjustments to harvest. We suggest that systems where fisheries differentially exploit phenotypically discrete, age‐invariant life histories provide excellent opportunities for detecting FIE.

## 1 INTRODUCTION

Fisheries impose artificial selection on exploited populations when harvest targets a nonrandom subset of phenotypes (Arnold & Wade, 1984; Heino, Pauli, & Dieckmann, 2015). For example, fisheries harvesting older, larger individuals impose selection favoring early maturation at smaller sizes (Olsen et al., 2004), while fisheries targeting individuals that arrive early to breeding grounds might impose selection favoring late‐arriving individuals (Mackey, McLean, & Quinn, 2001; Tillotson & Quinn, 2018). When traits subjected to fishery‐imposed selection have a heritable basis, then fisheries‐induced evolution (FIE) can result. There is now a compelling collection of case studies suggesting that FIE can occur in exploited wild fish populations (reviewed by Heino et al., 2015). These observational studies are supported by experimental work showing that fish life history traits such as growth rate and maturation age evolve rapidly under selection regimes relevant to those imposed by commercial fisheries (Biro & Post, 2008; Conover & Munch, 2002; Reznick & Ghalambor, 2005).

BOX 1 **Lessons from Louis**

My academic relationship with Louis began at the Canadian Society for Ecology and Evolution conference in 2009; I was asking him about the publication of a salmonid phylogeny that had been in the making for a while. A relatively decent French background, along with a blossoming understanding of phylogenetic analyses, led to my involvement in helping to publish the phylogeny that had to be repackaged from a thesis written in French. This was a four months postdoc with a single goal—to get the paper out while it was still relevant. Aside from the standard lessons that we all learn from gifted academics (i.e., do your best, get the work out and share it with the world), I learned a couple extra things about Louis during that time as well. The first was that he is up on the latest trends. The evidence for this came from my correspondence with Louis, always via email. In my email software program, the sign‐offs from Louis (on good news days) were always “Louis J”; this led me to think that he had a secret middle name that was kept off of the countless papers that he had authored. In retrospect, I’m glad that I never asked him about this—I first thought it was a weird recurring typo and attributed this to his remarkable ability to field emails at a rapid pace. Years (literally) later, I realized that the “J” was an early version of a poorly translate happy face emoticon, and neither my luddite brain nor my email program was equipped to make that translation. Bottom line: Louis was using emojis before it was something that became integral to virtual communication. The second lesson that I learned from Louis was that no matter how successful or driven a person may be, it is always okay to be human. Witnessing, and being a part of, Louis’ interactions has influenced how I interact with students and colleagues—Louis could be tough on people at times, but he was always compassionate in the end. I think about this every time I am a bit frustrated, and I remember how he always encouraged us in these weird moments, even if he was probably feeling a bit annoyed himself. To this day, over a decade after our short academic relationship, he still notices the littlest things about what I (and almost countless others) am up to; this is a testament to his humanity, and the care he puts into his trainees. That is the kind of mentor I strive to be. ‐Laura K. Weir.

While it is reasonable to assume that FIE can occur, for a number of reasons it is difficult to unequivocally conclude that observed changes in life history traits are the result of FIE (Heino et al., 2015). First, the strength of fishery‐imposed selection is often less than that imposed in relevant experiments (Hilborn & Minte‐Vera, 2008). Second, the nature of life history traits may constrain the evolutionary response to fishery‐imposed selection. Life history traits can have relatively low heritability because they are closely related to fitness, integrate variation across multiple component traits, are strongly affected by environmental variance, and may often be underlain by nonadditive dominance and epistatic variance (Merilä & Sheldon, 1999; Mousseau & Roff, 1987; Price & Schluter, 1991). Similarly, continuous life history traits such as age and size at maturity are mechanistically correlated, making it difficult to disentangle the contributions of phenotypic plasticity and evolutionary change to observed changes in trait values. While probabilistic maturation reaction norm analysis has gone some way in separating the contributions of plasticity and genetic adaption to changing maturation schedules, uncertainty is inevitable (Dieckmann & Heino, 2007). Third, many case studies of FIE involve single exploited populations or stocks monitored during a fishery and are thus unreplicated observations lacking experimental manipulation associated with the initiation or closure of the fishery (Heino et al., 2015). Finally, fisheries‐induced changes in life history traits can occur without FIE. Life history traits may change simply due to annual demographic adjustment to harvest or phenotypic plasticity in response to changes in population size, community composition, and environmental conditions caused by (or coincident with) the fishery of interest (Eikeset et al., 2016; Kuparinen & Merilä, 2007).

The challenge of implicating FIE may be best met using systems where: a fishery targets a subset of phenotypically discrete, age‐invariant, and heritable life histories; data on the relative frequency of each life history span periods before and after the initiation or cessation of the fishery; such data exist for multiple populations subjected to a common fishery; analyses can reasonably account for the effects of temporal variation in relevant population indices and environmental conditions (Kuparinen & Merilä, 2007).

**FIGURE 1:**
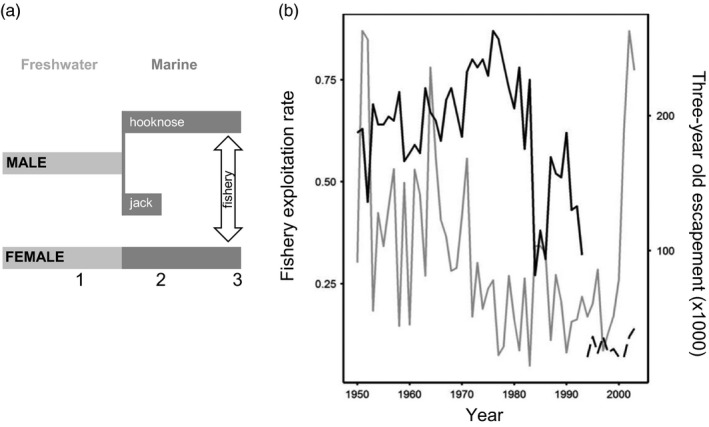
(a) Coho salmon life history on the Oregon (USA) coast. All fish spend 18 months rearing in freshwater before migrating to the ocean as smolts. All females and most males (hooknose) mature as three‐year‐olds after 18 months in the ocean, while some males (jack) mature precociously after six months at sea. A coast‐wide commercial fishery harvested three‐year‐old fish until its closure in 2003 (vertical arrow). (b) Estimates of the exploitation rate on three‐year‐olds (solid and dashed black lines) and coast‐wide three‐year‐old escapement (gray line) from 1950 to 2003. The commercial fishery was closed after the 1993 season, but harvest continued at low levels

Anadromous, semelparous Coho salmon (*Oncorhynchus kisutch*) from the Oregon coast (USA) meet these criteria reasonably well. All females and most males mature as three‐year‐olds following an 18‐month ocean phase, while some males mature precociously as smaller two‐year‐olds following six months in the ocean (Figure 1a). A coast‐wide commercial fishery targeted three‐year‐old males and females until 1993, when it was closed for conservation reasons (Figure 1a, b). Between 1950 and 2003, the Oregon Department of Fish and Wildlife (ODFW) conducted annual spawning ground surveys to monitor escapement for 30 populations; in 1981, a further 16 populations were added to the program. The effects of interannual variation in freshwater and marine conditions on Coho recruitment are well studied (e.g., Nickelson, 1986; Scarnecchia, 1981), and relevant environmental data are available from throughout the period of population monitoring.

Here, we use male Coho life history variation, the “pulse experiment” of a fishery and its closure, and demographic data from dozens of populations to test the prediction that fishery‐imposed selection against three‐year‐old males increased the proportion of two‐year‐old males in breeding populations (Gross, 1991; Myers, 1986). The data support this prediction and offer additional evidence that FIE contributed to the observed changes in male life history.

## 2 METHODS

### 2.1 Coho life history and FIE

Oregon coast Coho have two discrete, age‐invariant life histories. All juveniles spend 18 months in freshwater before migrating to the ocean as smolts, and all females mature as three‐year‐olds after 18 months in the ocean (Figure 1a). Most males also mature as three‐year‐old “hooknose” and typically adopt a “fighter” tactic when competing for access to spawning females. Other males mature precociously as “jacks” after six months in the ocean and usually adopt a “sneaker” tactic to fertilize eggs released by females mating with hooknose males (Gross, 1991). Male maturation age is heritable (jacks sire more jacks than hooknose males and vice versa), affected by maternal egg size (large eggs produce large juveniles more likely to mature as jacks), and condition‐dependent: juvenile males that reach a size/growth condition‐dependent threshold mature as two‐year‐old jacks (Appleby, Tipping, & Seidel, 2003; Iwamoto, Alexander, & Hershberger, 1984; Silverstein & Hershberger, 1992).

**FIGURE 2:**
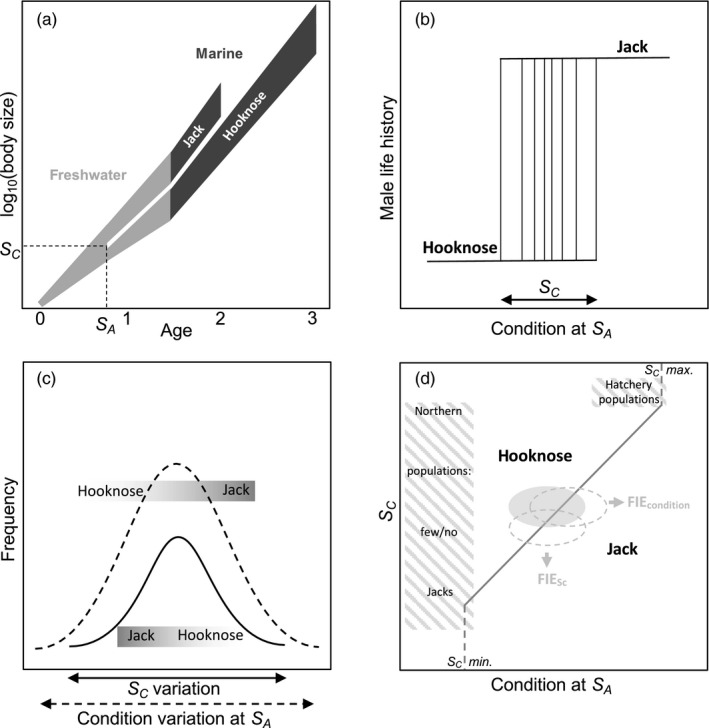
A graphical depiction of the evolutionary ecology and Fisheries‐Induced Evolution (FIE) of male Coho salmon life history (informed by Gross, 1991, 1996; Hutchings & Myers, 1994; Shuster & Wade, 2003; Tomkins & Hazel, 2007). (a) The two life histories of male Coho salmon from Oregon coast (USA) populations. After rearing for 18 months in freshwater, most males spend 18 months in the ocean before maturing as large three‐year‐old “hooknose,” while some males mature after only six months in the ocean as small two‐year‐old “jacks.” The irreversible decision between life history tactics occurs at age *S_A_* of the freshwater stage: juvenile males meeting a condition (size) threshold *S_C_* at age *S_A_* mature as jacks. Males adopting the hooknose and jack life history tactics typically use “fighting” and “sneaking” behavioral tactics, respectively, to gain access to spawning females. (b) A depiction of variation in male conditional life history strategy reaction norms. The life history tactic adopted by a male depends both on its heritable *S_C_* and its condition at age *S_A_*, which will depend on both environmental conditions and heritable variation in traits related to juvenile growth. (c) In any population, there will be variation in both *S_C_* and condition at age *S_A_*. The distributions are normal with greater variation in condition than *S_C_*. The graded bars highlight that the decision to adopt the jack strategy is more likely for males with high condition and/or a low *S_C_*. (d) The distributions of *S_C_* and condition at age *S_A_* can be visualized as an ellipse in a condition‐*S_C_* life history phase space. In this example, fish have condition‐*S_C_* combinations in both the hooknose (condition < *S_C_*) and jack (condition ≥ *S_C_*) regions of the phase space. The two dashed ellipses show how a fishery targeting hooknose males can drive FIE toward higher jack proportions by imposing viability selection against males with high *S_C_* and/or low condition. The orientation of an ellipse, and the evolutionary response to selection, will depend on the phenotypic and genetic correlations between condition and *S_C_*, which is assumed here to be zero. This life history phase space accommodates two important features of Coho “natural” history. First, jacks are rare or absent in northern populations with low juvenile growth rates because few or no males grow quickly enough to meet *S_C_ min.* at age *S_A_*. Second, hatchery populations that use only hooknose males for breeding regularly produce some jack males because unnaturally high juvenile growth rates allow some juvenile males to meet *S_C_ max.* at age *S_A_*

Understanding of the Coho jack‐hooknose system has developed alongside broader research on the evolutionary ecology of alternative reproductive phenotypes in both salmon and other animal taxa. Gross (1985) first described the system as a genetically polymorphic mixed evolutionarily stable strategy (mESS) in which population‐specific jack proportions occur where the lifetime fitness (survival to maturity × mating success) equality of the two strategies is maintained via negative frequency‐dependent sexual selection. He later proposed the system operates as a single genetically monomorphic conditional life history strategy with alternative tactics, the average lifetime fitnesses of which need not be equal (Gross, 1996). Neither of these models offers a satisfactory description of the jack‐hooknose system, and the latter has been criticized because the assumption of genetic monomorphism is unrealistic, especially in an evolutionary context (Shuster & Wade, 2003; Tomkins & Hazel, 2007). We build upon Tomkins and Hazel’s (2007) environmental threshold model, informed by research on other salmon species, to offer a conceptual framework for understanding the evolutionary ecology of the Coho jack‐hooknose system and the influence of FIE on jack proportions (Figure 2). Importantly, our framework accommodates two features of the system: in Coho and other salmon species precocious male maturation is rare or absent in northern populations where juvenile growth rates are low (Weir, Kindsvater, Young, & Reynolds, 2016); and jacks persistently occur in hatchery populations that use only hooknose males for breeding, but in which juvenile growth rates are markedly higher than in the wild (Vøllestad, Peterson, & Quinn, 2004).

The irreversible decision by a male to mature as a two‐year‐old jack or three‐year‐old hooknose is made at age *S_A_* during the early juvenile stage. Males that reach a size or condition threshold *S_C_* by age *S_A_* mature as jacks, while males that do not reach *S_C_* mature as hooknose (Figure 2a). Within a population, there is genetic variation in *S_C_*, such that, strictly speaking, each male's life history reaction norm is a different conditional strategy (Figure 2b). In any population, there is thus variation in both *S_C_* and condition at age *S_A_*, such that some proportions of males have condition ≥ *S_C_* and mature as jacks (Figure 2c). Males that mature as jacks will tend to have low *S_C_* and/or relatively high condition at age *S_A_* (Berejikian, Van Doornik, & Atkins, 2011). Whereas variance in *S_C_* is likely determined principally by genetic variance, the greater phenotypic variance in condition will depend on genetic variance in traits related to growth, but will also be strongly influenced by environmental variation. For any population, plotting the distributions of condition *S_A_* (*x*‐axis) and *S_C_* at age *S_A_* (*y*‐axis) creates an ellipse in a life history phase space (Figure 2d). Populations from the Oregon coast have ellipses spanning the hooknose to jack transition line (condition = *S_C_*), which extends from the minimum to maximum *S_C_* values of the individual life history reaction norms (Figure 2b, c). Wild populations with low juvenile growth rates fall on the left of the life history phase space, where few or no males reach the minimum *S_C_* required to mature as jacks. Hatchery populations with only hooknose broodstock and artificially high juvenile growth rates fall in the upper right corner, where some males achieve a condition greater than the maximum *S_C_* value.

For Coho salmon populations in the wild, a fishery that harvests hooknose males will impose artificial viability selection against fish with high *S_C_* and/or slow juvenile growth (Figure 2c). Such fisheries can thus drive FIE increases in jack proportion by increasing a population's mean condition at age *S_A_* and/or decreasing the population's mean *S_C_* at age *S_A_* (Figure 2d). This evolutionary response will be modified by the genetic architecture of and correlations between relevant traits (Merilä & Sheldon, 1999; Schluter, 1996), any environmentally induced changes in juvenile growth rates that affect the distribution of condition at age *S_A_*, and the effects on lifetime fitness of changes in density‐ and frequency‐dependent sexual selection acting on the mature male phenotypes (Berejikian et al., 2010; Fleming & Gross, 1994; Roff, 1996).

### 2.2 Demographic data

The ODFW began conducting spawning ground surveys during the peak of the Coho breeding season for 30 populations in 1950. In 1981, 16 populations were added to the monitoring program and multiple surveys were conducted throughout the breeding season for each population. The 46 populations were monitored annually until 2003. Though not every population was surveyed every year, these data provide estimates of three‐year‐old and jack abundance for dozens of populations during (1950, 1981–1993) and after (1994–2003) the commercial fishery. We considered only wild‐born fish, and the data were corrected for observer efficiency because during spawning ground surveys large adults are easier to see than smaller jacks; observer efficiency is corrected by multiplying the number of jacks by 2 and the number of adults by 1.3 (Solazzi, 1984). Assuming a 1:1 sex ratio for three‐year‐old adults (Koseki & Fleming, 2007; Nickelson, 2001; Young, 1999), annual jack proportion was calculated as:

$$\frac{\text{jack abundance}}{\text{jack abundance}+\text{hooknose abundance}}$$

Annual jack proportions were calculated using both the peak count and (from 1981) area under the curve (AUC) estimates. The AUC method uses data from the multiple surveys and the lifespan of spawning fish to calculate estimates of total escapement (Young, 1999). Jack proportion values based on peak counts and AUC estimates were strongly positively correlated (Table S1). We thus used peak count data in all analyses to include populations/years with insufficient surveys to calculate AUC estimates and to include data from 1950 to 1980. To estimate breeding densities (m^−2^), we used peak count data divided by the product of survey length and bankfull width, which we estimated according to Faustini, Kaufmann, and Herlihy (2009).

We calculated annual jack proportions using return year (jack and hooknose from year *t*) rather than brood year (jack *t*, hooknose *t* + 1) for three reasons (Koseki & Fleming, 2007). First, return year jack proportions reflect the conditions experienced by male Coho during breeding and are thus more relevant to sexual selection's role in determining within‐ and between‐population variation in the expression of male life history tactics. Second, using return year jack proportion controls for random and systematic errors associated with interannual variation in observer ability and survey conditions. Third, using brood year would reduce the number of observations available for analysis because a single year (*t*) of missing data eliminates three years (*t*−1, *t, t* + 1) of jack proportion estimates. However, we repeated our main analyses using brood year jack proportions, and our results were qualitatively unchanged (Figure S1).

### 2.3 Environmental data

Because the relative abundance of jacks and hooknose males may be influenced by temporal variation in environmental conditions, we compiled data on three factors known to affect survival and recruitment in Oregon coast Coho salmon: marine upwelling, sea surface temperature, and streamflow (Nickelson, 1986; Scarnecchia, 1981). While this list is not exhaustive, it did allow us to compare the environment experienced by jacks and adults during and after the fishery. The values for these environmental parameters fluctuated irregularly on a year‐to‐year basis (Figure S2). Our aim is not to assess correlates of jack and adult returns, but rather to control for how environmental conditions may affect the proportion of jacks in a given year. Thus, for each of these variables, we calculated the ratio of conditions experienced by jack (*t*) and hooknose (*t*−1) and compared these ratios before and after the fishery closure in 1994.

Marine upwelling data are mean monthly volume estimates obtained from the Pacific Fisheries Environmental Laboratory (http://www.pfeg.noaa.gov). Monthly volumes were obtained from March to September at three locations used by Oregon Coho (42°N, 125°W; 45°N, 125°W; 48°N, 125°W); these data were used as a proxy for food availability during the spring and summer of ocean entry. We summed marine upwelling volumes for the seven‐month period each year between 1949 and 2003. Beginning at 1950, we calculated the ratio of seasonal volumes experienced by jack and hooknose males during their first ocean year at each of the three locations as:

$$\text{Upwelling ratio}=\frac{\sum_{i=1}^{7}m_{t}^{3}}{\sum_{i=1}^{7}m_{t-1}^{3}}$$

where *m^3^* represents upwelling volume for a particular month, and *t* is a given year. In addition to analysis of upwelling ratios at individual stations, we calculated the mean of these three ratios as an overall metric for the area of the ocean used by Oregon Coho salmon. Because demographic data were collected for 30 populations beginning in 1950, with another 16 added in 1981 (see details below), we compared upwelling data before and after the fishery closure using all data as well as data only from 1981 onward, when all 46 populations could be used for other analyses. We found no strong evidence for differences between the mean upwelling ratios during and after the fishery (Table S2; Figure S2).

Sea surface temperature (SST) data were collected by the University Corporation for Atmospheric Research database (http://dss.ucar.edu). Monthly mean estimates for areas inside 42°N–45°N and 125°W–126°W from 1949–2003 were used in our analyses. Means between March and September were calculated for each year to reflect conditions during the spring and summer. As for the marine upwelling data, we took the ratio of SST means experienced by two‐year‐old jacks (µSST*_t_*) and three‐year‐old hooknose (µSST*_t_*_‐1_) males. Again, means during and after the fishery did not differ considerably for this variable (Table S2; Figure S2).

Streamflow data were obtained from the United States Geological Survey (USGS; http://waterdata.usgs.gov). We summed the mean monthly flow rate from five Oregon coast rivers between 1948 and 2003 and calculated the total flow experience by jack and hooknose males during their 18‐month freshwater residency as:

$$\text{Flow ratio}=\frac{\sum m^{3}s^{-1}{\text{Nov}}_{t-2}\;\text{to}\;{\text{May}}_{t}}{\sum m^{3}s^{-1}{\text{Nov}}_{t-3}\;\text{to}\;{\text{May}}_{t-1}}$$

where *t* is a given year. These values were compared as above and did not differ between the periods during and after the fishery (Table S2; Figure S2).

Although interannual variation in environmental conditions appeared unlikely to influence our results, we nonetheless constructed additive models to account for the effect of marine upwelling, sea surface temperature, and streamflow on jack proportions. For each population, jack proportion was regressed on the ratios of the three environmental parameters using a linear model:

$$Y_{i}=\alpha +\beta_{1}\left(\text{upwelling ratio}\right)+\beta_{2}\left(\text{sst ratio}\right)+\beta_{3}\left(\text{flow ratio}\right)+\varepsilon_{i}$$

where *Y_i_* is a population's jack proportion for year *i.* We used the residuals from this model for analyses of “environmentally corrected” jack proportions.

### 2.4 Statistical analyses

#### 2.4.1 Pre‐ and postfishery closure comparison

To test our main prediction that fishery‐imposed selection against hooknose males would drive increases in jack proportions (Figure 2d), we used data from 1981 to 1993 and 1994 to 2003 to calculate the mean jack proportions of 46 populations during and after the fishery. The same approach was used for the environmentally corrected data; residuals from the linear model for each population were averaged for the two time periods. Jack proportion during and after the fishery for both datasets were compared using linear mixed‐effects models with stream as a random effect to account for the paired nature of the data; the unit of observation for this analysis was the 46 populations.

#### 2.4.2 Evidence for evolutionary change

For given numbers of returning hooknose and jack males, harvesting three‐year‐old fish will clearly increase return year jack proportions through demographic adjustment. To assess the relative importance of demographic adjustment and FIE, we conducted four analyses exploring interannual variation in populations’ jack proportions during the fishery. For three “temporal” hypotheses, we used only the 30 populations with data from 1950 because these populations provide a sufficient number of observations for meaningful analyses. Before doing these analyses, we interrogated the data to determine whether temporal autocorrelation affected our analyses. We found no evidence of temporal autocorrelation (Appendix S1: Methods and Analyses) and thus proceeded with mixed modeling and correlation approaches.

First, we tested whether fishery exploitation rate significantly affected interannual variation in jack proportion. To this end, we calculated the correlation coefficient between exploitation rate and jack proportion for each of the 30 populations; positive correlations would suggest demographic adjustment to harvest was important. We then tested whether exploitation rate affected interannual variation in jack proportion by regressing jack proportion on exploitation rate using a linear mixed‐effects model with jack proportion as the dependent variable, exploitation rate as a fixed effect, and population as a random effect (*n* = 30). Second, noting that exploitation rate did not increase systematically during the fishery (Figure 1b), we used a linear mixed‐effects model to regress jack proportion on time, again using population as a random effect (*n* = 30) to determine whether there was a tendency for jack proportions to increase through time, a pattern that is consistent with an evolutionary response to selection against hooknose males. Third, we quantified the relationship between population‐level temporal changes in jack proportion and adult density during the fishery to test whether the rate of increase in jack proportion was related to the rate of decline in adult escapement across populations. Such a relationship would implicate demographic adjustment as an important mechanism in driving temporal changes in jack proportion. For this analysis, we calculated the slope of change in jack proportion and adult density for each population from 1950 to 1993 and tested the correlation between these values (*n* = 30).

Finally, using data for all 46 populations between 1981 and 2003, we explored how the fishery affected the relationship between three‐year‐old breeding density (escapement) and the proportion of males adopting the jack tactic. If postfishery declines in jack proportions were due simply to demographic adjustment, we expect both adult and jack escapement to increase, but jack escapement to increase by less. If such declines reflect evolutionary change, we expect the relationship between adult escapement (i.e., the number of eggs deposited and fry produced) and jack escapement to change; there should be fewer jacks per spawning female. We explored these complementary predictions two ways. For each of the 46 populations in the 1981 to 2003 dataset, we calculated mean adult escapement (number/m^2^), mean jack escapement, and mean jack proportion during (1981–1993) and after (1994–2003) the fishery. We first used a linear mixed‐effects model with mean jack proportion (untransformed) as the response variable, period as a fixed‐effect class variable, mean three‐year‐old breeding density as a continuous variable, and population as a random effect to account for the paired nature of the analysis. We asked whether declines in jack proportion following the fishery closure were due simply to increases in three‐year‐old density (same slope and intercept) or to a change in the relationship between three‐year‐old density and the proportion of males adopting the jack tactic (different slopes and/or intercepts). Second, we compared mean adult and jack escapement across populations during and after the fishery using linear mixed‐effects models with population as a random effect (*n* = 46). If declines in jack proportion reflected an evolutionary response to the cessation of the fishery targeting adults, we predict that jack escapement would decline despite increases in adult escapement.

All analyses were conducted in R 3.6.2 (R Core Team, 2019). Mixed models were constructed using the lme4 package in R (Bates, Mächler, Bolker, & Walker, 2015). To assess model fits, likelihoods were calculated using the maximum likelihood method and compared using Akaike information criteria (AIC).

## 3 RESULTS

**FIGURE 3:**
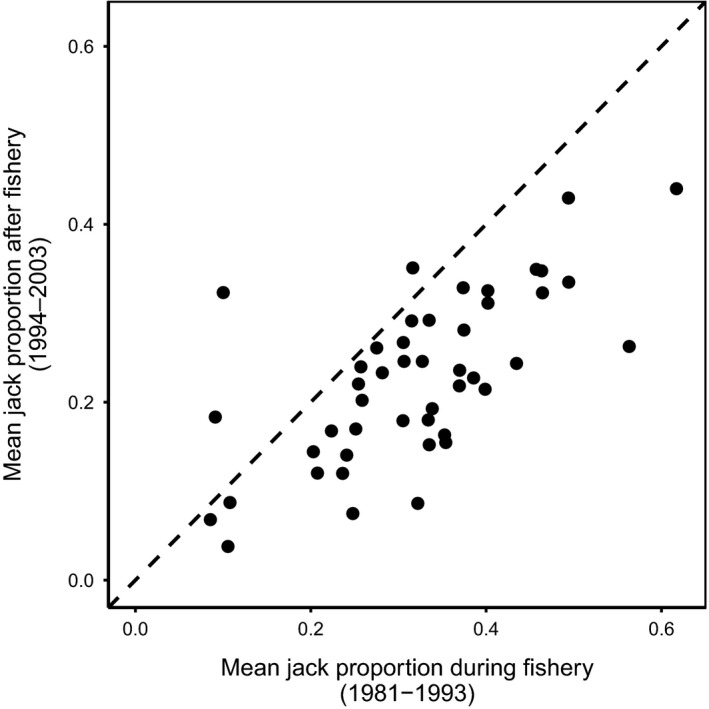
In 43 of 46 populations, mean jack proportion was higher in the 13 years during the fishery than in the 10 years following the fishery closure. The dashed line is 1:1

**FIGURE 4:**
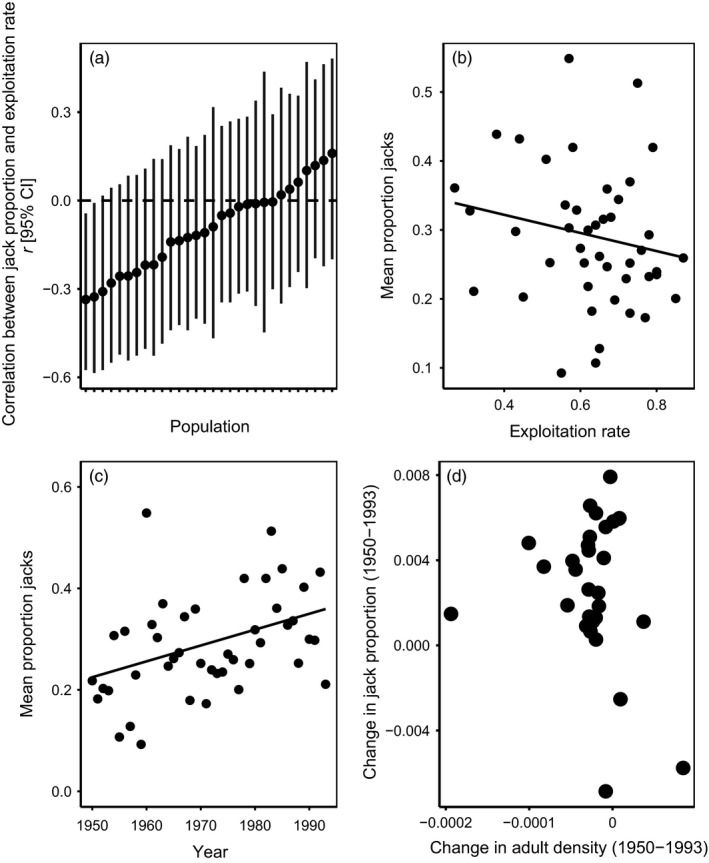
(a) For the 30 populations with data from 1950, there was no tendency for interannual variation in fishery exploitation rate to be positively correlated with interannual variation in jack proportion between 1950 and 1993. (b) Jack proportion was not related to exploitation rate from 1950 to 1993. (c) Jack proportion increased gradually over time during the fishery. (d) Jack proportion increased between 1950 and 1993 in 27 of the 30 populations, and the rate of increase in jack proportion was unrelated to changes in three‐year old density during the fishery

We found strong support for our principal prediction. In 43 of 46 populations, mean jack proportion was higher during than after the fishery (Table 1; Figure 3). For the 30 populations with data from 1950, there was no evidence that exploitation rate and jack proportion were positively correlated during the fishery; rather, most correlations were negative (Figure 4a). A linear mixed‐effects model that directly examined the influence of exploitation rate on jack proportion supports these results, such that the overall effect of harvest rate on jack proportion across all populations was weak and negative (Table 1, Figure 4b). While exploitation decreased following its peak in the 1970s (quadratic model *F*_2, 41_ = 15.07, *R* ^2^ = 0.42; Figure 1b), jack proportion increased gradually through time across all populations (Table 1; Figure 4c). At the population level, jack proportion increased through time in 27 of the 30 populations (Figure 4d). Among these same 30 populations, we found little evidence that increases in jack proportion were directly related to declines in adult density (r [95%CI] = −0.21 [−0.53,0.16]; Figure 4d). Taken together, these results suggest that during the fishery jack, proportions were: (a) higher than after its closure (Figure 3); (b) gradually increasing through time (Figure 4c,d); and (c) were not strongly dependent on interannual variation in exploitation rate or changes in adult density (Figure 4a,b,d). Thus, our main and supplementary results reveal that fishery‐imposed selection was associated with increased jack proportions across dozens of populations and that demographic adjustment to variation in exploitation rate does not appear to solely explain variation in jack proportion among or across populations. These results were qualitatively unchanged after controlling for interannual variation in environmental conditions.

**TABLE 1:** Model fits for linear mixed‐effects models to examine evidence for evolutionary change

Model parameter	k	AIC	ΔAIC	ω_i_	Parameter estimate (± *SE*)
Comparison between pre‐ and postfishery jack proportions					
Fishery	**4**	**−170.51**	**0**	**1**	−0.093 ± 0.012
(intercept)	3	−137.45	33.06	0	
Relationship between jack proportion and harvest rate					
Harvest rate	**4**	**−107.71**	**0**	**0.98**	−0.15 ± 0.05
(intercept)	3	−99.82	7.89	0.02	
Change in jack proportion over time during fishery					
Year	**4**	**−127.56**	**0**	**1**	0.0029 ± 0.00052
(intercept)	3	−99.52	27.74	0	
Relationship between jack proportion and the interaction between adult density and fishery					
Fishery	**4**	**−170.51**	**0**	**0.44**	
Adult density + fishery	**5**	**−170.10**	**0.41**	**0.36**	
Adult density × fishery	**6**	**−168.94**	**1.56**	**0.20**	
(intercept)	3	−137.45	33.06	0	
Adult density	4	−135.53	34.98	0	
Comparison between pre‐ and postfishery adult densities					
Fishery	**4**	**−1025.19**	**0**	0.99	0.00050 ± 0.00013
(intercept)	3	−1015.17	10.03	0.01	
Comparison between pre‐ and postfishery jack densities					
Fishery	**4**	−1236.70	**0**	0.54	−0.000054 ± 0.000035
(intercept)	3	−1236.39	0.31	0.46	

Note

The number of parameters (k), Akaike information criterion values (AIC), the difference between the best model and the other model (ΔAIC) and relative model weight (ω_i_) are shown for each analysis. We considered the model with the lowest AIC to be of best fit if ΔAIC > 2; these models are in bold. Parameter estimates are shown if they provide information regarding the direction of change in the data.

**FIGURE 5:**
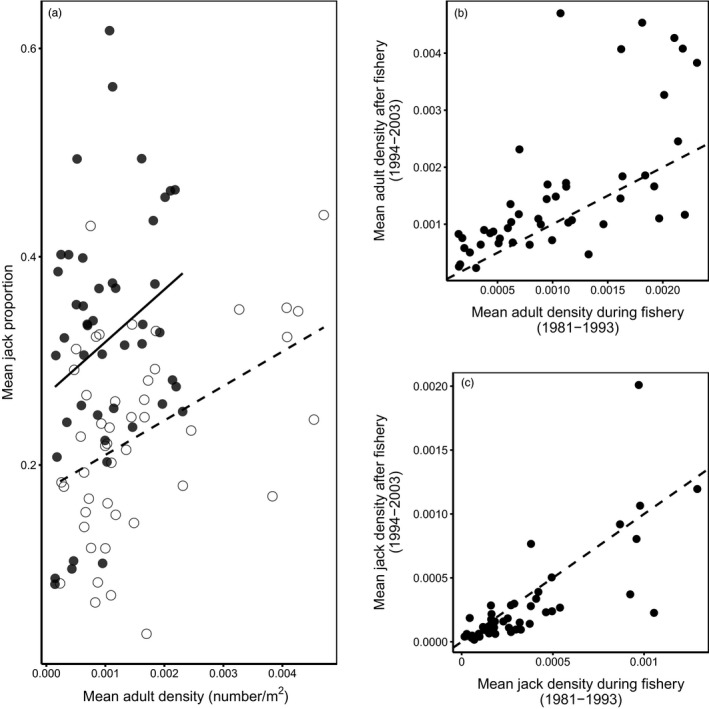
(a) Mean adult densities and jack proportions of 46 populations during (1981–1993; filled symbols, solid line) and after (1994–2003; open symbols, dashed line) the fishery targeting three‐year‐old adults. (b) Adult density (m^−2^) during and after the fishery. Adult densities increased significantly after the fishery closure. (c) Jack density (m^−2^) during and after the fishery. Jack densities tended to decrease after the fishery closure. The dashed lines in B and C are 1:1.

Our final analysis using the 46 populations with data from 1981 reveals how the relationship between adult density, jack density, and jack proportion was affected by the fishery (Figure 5). During both periods, populations with higher mean adult breeding densities tended to have higher mean jack proportions, a pattern consistent with previous observations (Young, 1999). While the slopes of these relationships were similar, the intercept was significantly higher during the fishery (Table 1); thus, for a given density of three‐year‐old spawners, the proportion of males adopting the jack life history tactic was significantly higher during than after the fishery. This result is not simply due to adult densities increasing more than jack densities following the fishery closure. While adult densities increased significantly following the 1993 closure (Table 1; Figure 5b), jack densities tended to decrease (Table 1; Figure 5c).

## 4 DISCUSSION

Data on male life histories from 46 Coho salmon populations provide compelling evidence for FIE. A fishery targeting three‐year‐old males adopting the hooknose‐fighter tactic imposed artificial viability selection favoring two‐year‐old males adopting the jack‐sneaker tactic. Consistent with the predicted evolutionary response (Figure 2d), a striking majority of populations had higher jack proportions during than after the fishery. Clearly, our data are observational and lack control populations not subjected to fishery exploitation, making it impossible to unequivocally implicate FIE. Furthermore, harvesting hooknose males must strictly increase return year jack proportions through within‐generation demographic adjustment. Still, demographic adjustment alone appears an insufficient explanation for our main result. First, jack proportions tended to increase through time during the fishery despite no increase in exploitation rate. Second, at the population level there was no tendency for positive correlations between interannual variation in jack proportion and exploitation rate. Third, across all populations mean jack proportion was unrelated to exploitation rate. Fourth, there was no clear relationship between declines in adult density and increases in jack proportion during the fishery. Finally, while adult densities increased markedly following the fishery closure, jack densities decreased. All of these results held when controlling for variation in environmental conditions known to affect Coho salmon population dynamics. Within this context, we discuss relevant evolutionary and ecological processes in turn.

There are two nonexclusive direct mechanisms by which populations’ jack proportions might increase through FIE. Fishery‐imposed viability selection against hooknose males might favor juveniles with lower‐threshold reaction norms (*S_C_* in Figure 2b,c), resulting in downward shifts in the distributions of *S_C_*, and higher proportions of males meeting threshold conditions for adopting the jack life history (FIE_Sc_ in Figure 2d). Alternatively, such selection might favor faster‐growing, higher‐condition juveniles (Figure 2c), drive an upward shift in the distributions of condition, again increasing the proportions of males achieving *S_C_* and maturing as jacks (FIE_condition_ in Figure 2d).

The roles of these two direct mechanisms in driving FIE are unclear and likely complex. Data from Coho (Silverstein & Hershberger, 1992, 1994), Chinook (*Oncorhynchus tshawytscha*; Spangenberg et al., 2015), and Atlantic salmon (*Salmo salar*; Aubin‐Horth & Dodson, 2004; Piché, Hutchings, & Blanchard, 2008) suggest there is heritable variation in both juvenile growth rate (condition) and *S_C_* within and between our study populations (Figure 2b,c). The environmental threshold model (Tomkins & Hazel, 2007) sensibly assumes higher phenotypic variance in condition than *S_C_* (Figure 2c), but how the trait distributions respond to viability selection against hooknose males will depend on the heritability of the traits, and the genetic architecture of (Merilä & Sheldon, 1999) and correlations between (Schluter, 1996) traits. We are not aware of data on how *S_C_* variance might depend on additive, dominance, and epistatic genetic variance, but condition variance in nature is likely strongly dependent on environmental variance, which would reduce its heritability. Our heuristic model of FIE (Figure 2d) assumes condition and *S_C_* are uncorrelated and normally distributed, which may not be realistic. For example, the phenotypic/genetic correlation between the traits in natural populations would be negative if hooknose juveniles tend to grow slowly and have high *S_C_* values and the opposite holds for jack juveniles (Figure 2c). In this case, wild population ellipses would be tilted downward to the right in the life history phase space (Figure 2d), and the rate and direction FIE would progress along genetic lines of least resistance as determined by the genetic variances of and covariance between condition and *S_C_* (Schluter, 1996).

We should expect the evolutionary response to direct viability selection against hooknose males to be mediated by other evolutionary and ecological processes (Eikeset et al., 2016; Gross, 1991; Kuparinen & Merilä, 2007). From an evolutionary perspective, fishery‐imposed selection favors jacks directly through the survival component of lifetime fitness, but the demographic consequences of fishery harvest are likely to indirectly affect the relative reproductive fitness of hooknose and jack males. First, the mean mating success of the jack‐sneaking tactic is expected to decline with jack proportion through negative frequency‐dependent sexual selection (Gross, 1985; Hutchings & Myers, 1988) as mediated by habitat conditions (DeFilippo et al., 2018). Second, changes in the form and strength of sexual selection likely favor hooknose males as breeding densities decline due to harvest. The jack life history is expected to be favored at high breeding densities because sneaking tactics are favored at high breeding densities in general (Roff, 1996) and because sexual selection on male body size in Coho changes from disruptive to directional to as breeding density declines (Fleming & Gross, 1994). Thus, the indirect effects of frequency‐ and/or density‐dependent sexual selection are expected to favor hooknose males and act in the opposite direction to the effects of fishery‐induced viability selection favoring jacks.

The fishery targeting hooknose males may also have affected jack proportions through ecological processes other than simple demographic adjustment. Fishery harvest reduced breeding densities (Figures 1b and 5b), and thus juvenile densities, which is expected to increase juvenile growth rates, conditions at age *S_A_*, and the proportion of males meeting condition‐dependent thresholds for maturing as jacks (Grant & Imre, 2005; Rosenfeld, Leiter, Lindner, & Rothman, 2005; Vincenzi, Satterthwaite, & Mangel, 2012; Figure 2c). Alternatively, reduced escapement might lower juvenile growth rates by reducing levels of carcass‐derived nutrients in streams (Heintz et al., 2004). While such ecological processes likely operate alongside and mediate any FIE changes in jack proportions, it is noteworthy that high breeding densities are associated with high jack proportions (Young, 1999, Figure 5a). This may be because density‐dependent sexual selection favoring jacks outweighs the effects of density‐dependent reductions in juvenile growth. Also, the effect of intraspecific asymmetric competition tends not reduce growth rates of large, dominant juveniles destined to mature as jacks (Rosenfeld et al., 2005). The number and complexity of such processes make it unsurprising that we found no relationship between declines in adult density and increases in jack proportions during the fishery (Figure 4b).

We conclude that the data from Oregon coast Coho salmon populations offer compelling evidence for FIE. Identifying the specific evolutionary mechanism and quantifying the relative importance of FIE and ecological processes remain open challenges. Such uncertainty underscores the challenge of studying FIE in nature. Notwithstanding these caveats, our study highlights the value of using the discrete male life history tactics common in anadromous salmonids to study FIE (Fleming & Reynolds, 2004; Weir et al., 2016). Indeed, a recent study of a single exploited Sockeye salmon (*O. nerka*) population revealed the same temporal increase in jack proportion observed across Oregon coast Coho populations (DeFilippo et al., 2019). We encourage others to compile and interrogate similar datasets.

## Supporting information

Appendix S1

## Data Availability

The data herein are available upon request from the Oregon Department of Fish and Wildlife.
