# Supplementary material for: Fisheries‐induced evolution of alternative male life history tactics in Coho salmon
Source: Evol Appl. 2020 Apr 21;13(6):1501–12. doi: 10.1111/eva.12970 (PMC7359830; doi:10.1111/eva.12970)

Table S1. Correlations between jack frequency calculated from peak count estimates and AUC estimates for each of 46 streams for each year beginning in 1981.

stream	r [CI]	n
1	0.95 [0.86, 0.98]	17
2	0.86 [0.68, 0.95]	18
3	0.84 [0.61, 0.94]	15
4	0.75 [0.46, 0.9]	18
5	0.98 [0.94, 0.99]	15
6	0.96 [0.90, 0.99]	16
7	0.97 [0.93, 0.99]	17
8	0.94 [0.86, 0.98]	18
9	0.94 [0.8, 0.98]	11
10	0.97 [0.92, 0.99]	17
11	0.99 [0.98, 1.00]	14
12	0.94 [0.86, 0.97]	20
13	0.81 [0.53, 0.93]	14
14	0.99 [0.97, 1.00]	8
15	0.68 [0.36, 0.86]	20
16	0.36 [-0.14, 0.71]	16
17	0.94 [0.87, 0.98]	21
18	0.55 [0.17, 0.79]	20
19	0.74 [0.46, 0.89]	20
20	0.88 [0.73, 0.95]	20
21	0.87 [0.71, 0.95]	18
22	0.68 [0.32, 0.87]	17
23	0.94 [0.83, 0.98]	16
24	0.98 [0.96, 0.99]	17
25	0.96 [0.91, 0.99]	18
26	0.86 [0.69, 0.94]	21
27	0.83 [0.62, 0.93]	19
28	0.74 [0.46, 0.89]	20
29	0.92 [0.81, 0.97]	20
30	0.91 [0.78, 0.96]	19
31	0.96 [0.89, 0.98]	17
32	0.90 [0.77, 0.96]	18
33	0.82 [0.58, 0.93]	17
34	0.90 [0.76, 0.96]	17
35	0.94 [0.84, 0.98]	16
36	0.86 [0.68, 0.94]	20
37	0.47 [-0.08, 0.8]	12
38	0.74 [0.42, 0.90]	16
39	0.93 [0.83, 0.98]	17
40	0.94 [0.85, 0.98]	19
41	0.96 [0.90, 0.99]	17
42	0.88 [0.67, 0.96]	13
43	0.93 [0.84, 0.97]	19
44	0.92 [0.79, 0.97]	16
45	0.90 [0.75, 0.96]	18
46	0.83 [0.58, 0.94]	14
mean	0.86 [0.69, 0.94]	

Effects of brood year and data inclusion on the main result

Our main analysis includes all 46 populations, using data from 1981 onward to maintain a balanced design. However, we recognize that this may influence the overall result, and thus we have done some supplementary analysis to examine whether this pattern has changed over time. Similarly, because changes may manifest in brood years rather than within counts for a given year, we have also calculated jack proportion based on brood year, and re-created figure 3 using these data (Fig S1). The result from these analyses are below.

Table S2. Model comparisons of the best fit model that included fishery, to a model with only an intercept. ΔAICc is the difference between these two models; ω_i is the weight of the best model that includes fishery as a factor compared to all two possible models.

**Jack proportion based on:**	**ΔAICc**	**ω_i**
**Annual counts**		
all available data from 46 populations	10.7	1.0
all available data from 30 populations that have been censused since 1950	8.43	0.99
**Brood years**		
all available data from 46 populations	10.66	1
all available data from 30 populations that have been censused since 1950	10.42	0.99

Table S3. Summary of linear mixed effects models comparing the ratio of environmental conditions experienced by jacks and adults over time. ΔAIC is the difference between the best model and the other model, while ω_i is the relative weight of the models. In all cases, the models that included only and intercept (the mean) and those that included fishery were equivocal, of the intercept-only model was a slightly better fit to the data.

		**Models including data from 1950-2003**		**Models including data from 1981-2003**	
**variable**					
		**ΔAIC**	***ωi***	**ΔAIC**	***ωi***
upwelling					
latitude 42	(intercept)	0	0.7	0	0.71
	fishery	1.74	0.3	1.83	0.29
latitude 45	(intercept)	0	0.54	0	0.65
	fishery	0.31	0.46	1.27	0.35
latitude 48	(intercept)	0	0.71	0	0.73
	fishery	1.81	0.29	1.99	0.27
mean upwelling	(intercept)	0	0.60	0	0.64
	fishery	0.78	0.40	1.18	0.36
sea surface temperature	(intercept)	0	0.71	0	0.72
	fishery	1.81	0.29	1.85	0.28
streamflow	(intercept)	0	0.57	0	0.66
	fishery	0.57	0.43	1.29	0.34

Methods for detecting temporal autocorrelation

We examined our data to assess whether patterns of change were associated with autocorrelative effects (i.e., years with many animals resulting in subsequent years with many animals, or vice versa). We expect that lags of 2-3 years to be of most concern, along with obvious patterns of increase and decrease. However, given that these are annual counts, we do not necessarily expect that there will be strong autocorrelative effects between data points.

To examine autocorrelation, we first plotted yearly autocorrelations using the acf function in R. This procedure was done for each population, as there may be differences among populations in this parameter. Our examination of these plots for both jack proportion using counts on spawning grounds within years, as well as jack proportions for animals from the same brood year, revealed that autocorrelation is weak in most populations, particularly for the lag periods of interest (Figure S3).

Further to these analyses, we also constructed full mixed models, both with and without an autocorrelative parameter, to compare the model fits. These models included year and fishery as fixed effects, and stream as a random effect with a random intercept. The autoregressive parameter was corCAR1, as this is most appropriate for time series data with missing values. This parameter took the form ~year. Model fit, assessed by AICc, with these full temporal trends for the 46 populations are shown in Table S3. Removing the autocorrelative parameter improved the model fit slightly, indicating that it is unlikely to strongly influence the patterns that we observed in our data.

Table S4. AICc values for mixed model effects comparing the influence of year and fishery on jack proportions (both within year and within brood years), with and without the autoregressie (AR) parameter. We also include φ values, which are estimates of the correlation between successive data points for each model.

	**Jack proportions within year**			**Jack proportions within brood year**		
	AR parameter included	φ	No AR parameter	AR parameter included	φ	No AR parameter
year ×fishery	-289.09	4.30×10^{-8}	-291.10	290.04	3.33×10^{-9}	288.02
year+fishery	-289.50	4.25×10^{-8}	-291.51	298.58	1.62×10^{-9}	296.56
fishery	-264.89	0.016	-266.51	300.39	2.61×10^{-9}	298.38
year	-248.80	0.030	-249.45	313.67	2.30×10^{-9}	311.66
(intercept)	-249.76	0.031	-250.31	311.88	2.93×10^{-9}	309.88

Fig S1. Mean jack proportion before and after the fishery based on brood years.

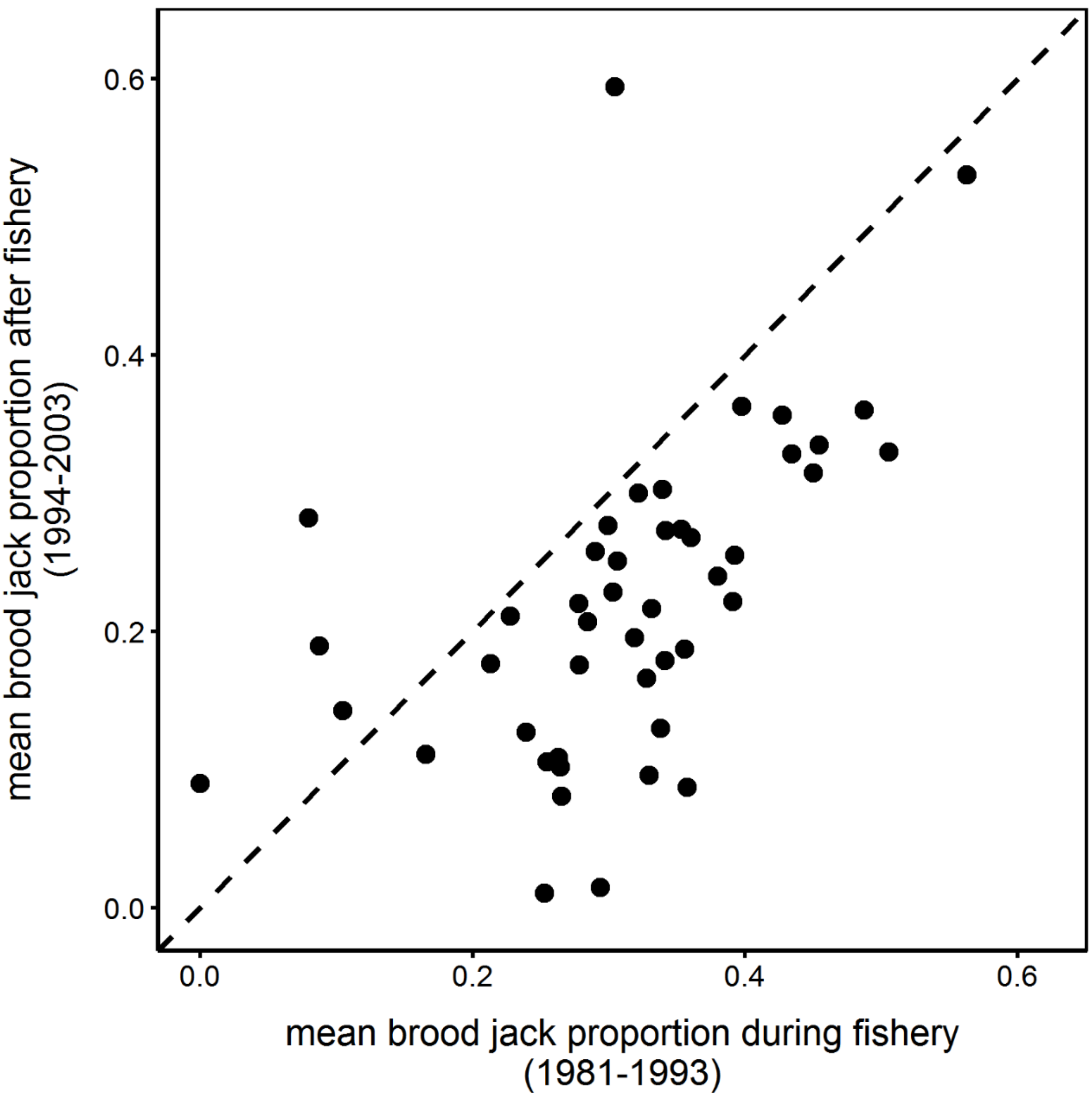

Fig. S2 Temporal patterns for A) marine upwelling, B) sea surface temperature, C) streamflow rate. Solid lines are means during the fishery, and dashed lines are means after the fishery was closed.

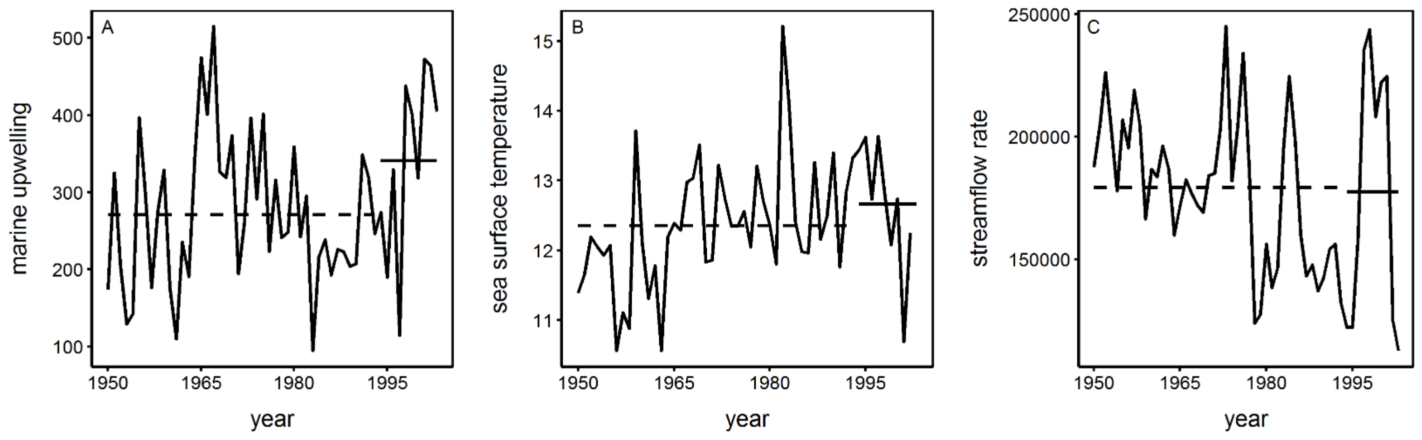

Fig S3. ACF values over lag times for jack proportions within for 46 populations. The x- axis is the lag, and the y-axis is the ACF value. Dashed blue lines are cut-off correlation values, above or below which correlations may be deemed significant.

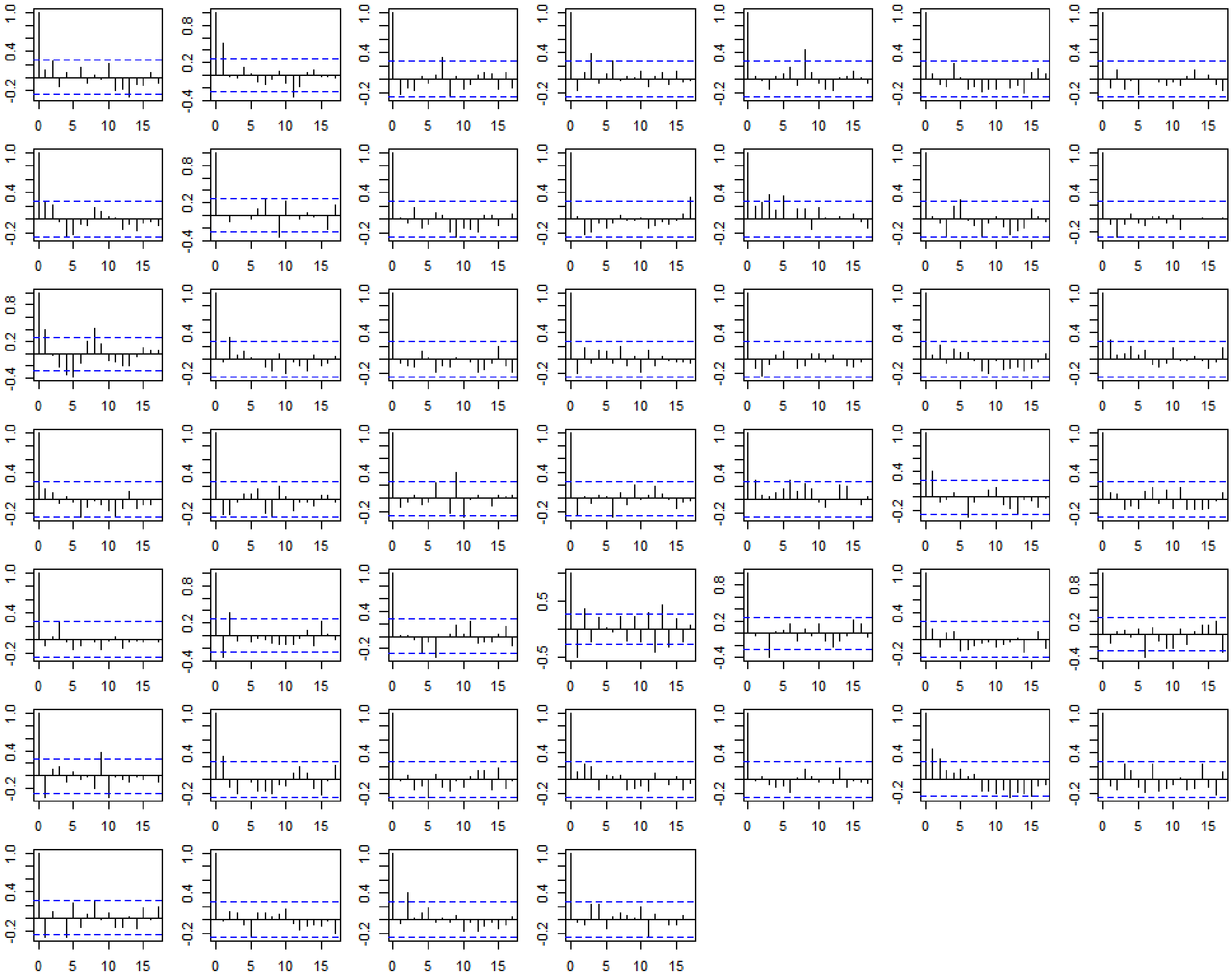

Fig S4. ACF values over lag times for jack proportions within brood for 46 populations. The x- axis is the lag, and the y-axis is the ACF value. Dashed blue lines are cut-off correlation values, above or below which correlations may be deemed significant.

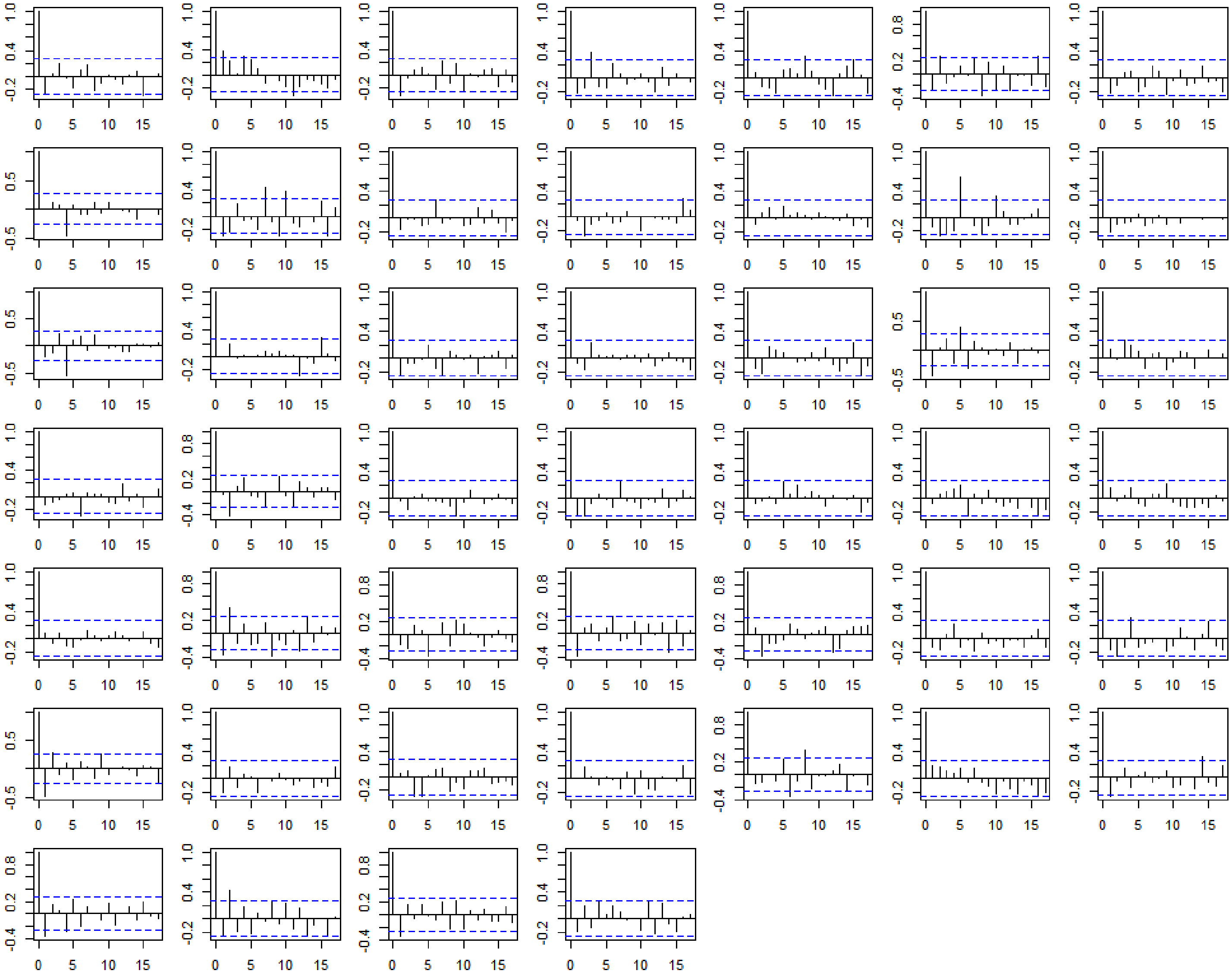

Supplement: Supplementary file 1 — Appendix S1 [file EVA-13-1501-s001.pdf]
